# Inosine Prevents Colorectal Cancer Progression by Inducing M1 Phenotypic Polarization of Macrophages

**DOI:** 10.3390/molecules30010123

**Published:** 2024-12-31

**Authors:** Yuchen Ma, Xiaoli Qian, Qun Yu, Yadan Dong, Jiapeng Wang, Heng Liu, Huai Xiao

**Affiliations:** Yunnan Provincial Key Laboratory of Entomological Biopharmaceutical R&D, National-Local Joint Engineering Research Center of Entomoceutics, College of Pharmacy, Dali University, Dali 671000, China; mayuchen95@163.com (Y.M.); qianxl20210513@163.com (X.Q.);

**Keywords:** inosine, macrophage polarization, colorectal cancer

## Abstract

Inosine (IS) is a naturally occurring metabolite of adenosine with potent immunomodulatory effects. This study investigates the immunomodulatory effects of inosine, particularly its ability to inhibit the development of colorectal cancer (CRC) cells CT26 through modulation of macrophage phenotypes. Aside from the already reported effects of inosine on T cells, in this study, in vitro experiments revealed that inosine could modulate macrophage phenotype. The effects of inosine on the M1/M2 macrophage polarization were investigated at the cellular level. Its role in regulating CRC proliferation and migration was further examined. In addition, a CT26 tumor mouse model was established to assess the mechanism of action of inosine by tumor weight measurement, immunohistochemistry, and immunofluorescence. Inosine significantly increased M1 macrophage markers CD86 and iNOS and enhanced the anti-tumor activity of M1 macrophages, effectively inhibiting CRC progression and metastasis potential. In vivo, inosine had significant tumor inhibitory activity. It also significantly reduced the expression of Ki-67 and promoted the polarization of M1 macrophages.

## 1. Introduction

Malignant tumors have become one of the most significant threats to global health over the past half-century [1]. Among these, colorectal cancer (CRC) is a prevalent and lethal malignancy of the digestive system, posing a serious risk to human health and life [1]. The global incidence and mortality rates of CRC are steadily rising [2]. According to the “Global Cancer Data Statistics 2022”, CRC accounts for 10.0% of all cancer cases and 9.4% of cancer-related deaths, making it the second most lethal cancer after lung cancer [3]. This growing burden has placed considerable medical and economic stress on societies worldwide [4]. Furthermore, CRC exhibits a high propensity for metastasis, with approximately 90% of patient deaths resulting from metastatic disease [5,6].

CRC arises from malignant lesions in the colonic mucosal epithelium or glands, driven by various oncogenic factors, including environmental and genetic influences [7]. It is a heterogeneous disease characterized by multiple pathogenic mechanisms such as somatic mutations, gene fusions, genetic instability, and epigenetic changes [7]. Current therapeutic approaches, including surgery, chemotherapy, and radiotherapy, face significant challenges, including severe side effects, drug resistance, and toxicity [8]. As drug therapy remains the cornerstone of cancer treatment, the development of new anti-cancer drugs has become a hotspot of drug research and development in recent years. There is a growing demand for novel therapeutic strategies that are both effective and less toxic [9].

The tumor microenvironment (TME) plays a pivotal role in tumor metastasis, immune suppression, and resistance to chemotherapy, with increasing attention being directed toward immunomodulatory interventions as a potential therapeutic avenue [10]. The TME comprises various cellular components, including tumor cells, infiltrating immune cells (macrophages, lymphocytes, dendritic cells, etc.), and mesenchymal cells (cancer-associated fibroblasts, endothelial cells, etc.) [11,12]. Among these, tumor-associated macrophages (TAMs) are the most abundant immune cells infiltrating the TME and play a crucial role in promoting tumor development [13]. TAMs facilitate tumor growth and invasion through the secretion of cytokines such as vascular endothelial growth factor (VEGF) and matrix metalloproteinases (MMPs), which induce angiogenesis at the tumor site [14]. Additionally, TAMs support tumor growth and invasion by releasing prostaglandin E2 (PGE2) and transforming growth factor β (TGF-β). Other immunomodulatory factors such as interleukin-10 (IL-10) inhibit the cytotoxic activity of T lymphocytes and natural killer (NK) cells to exert an immunosuppressive effect [15]. This suggests that TAMs are potential targets for tumor therapy.

Macrophages exhibit remarkable plasticity and can adapt their phenotype and function according to environmental cues, contributing to tumor progression [16]. They are broadly classified into classically activated (M1) and alternatively activated (M2) macrophages [16]. M1 macrophages, induced by interferon-gamma (IFN-γ) and lipopolysaccharide (LPS), are pro-inflammatory and exhibit anti-tumor properties, marked by the production of IL-12, tumor necrosis factor-alpha (TNF-α), and inducible nitric oxide synthase (iNOS) [17]. Conversely, M2 macrophages, polarized by IL-4, are associated with tissue repair, anti-inflammatory responses, and tumor promotion, marked by arginase (ARG-1), IL-10, TGF-β, and the mannose receptor (CD206) [15,18,19]. In the TME, TAMs predominantly display an M2 phenotype, fostering tumor growth and immune suppression. However, a small population of TAMs retains the M1 phenotype, which possesses anti-tumor potential [20]. Understanding the factors regulating macrophage polarization could offer novel targets for cancer therapy [20]. Recent therapeutic strategies aim to reprogram TAMs toward the M1 phenotype or inhibit the M2 phenotype to reduce tumor progression, making TAM polarization a potential prognostic indicator and therapeutic target [21].

Inosine (IS), a naturally occurring purine nucleoside, is involved in metabolic pathways and energy production [22]. It is widely found in plants and animals and has been used clinically as an adjuvant in the treatment of chronic hepatitis and other diseases due to its favorable safety profile [22]. Recent studies have demonstrated that in vivo inosine and its derivatives could inhibit the activity of ubiquitin-activating enzyme 6(UBA6) to increase the immunogenicity of tumor cells, improving the tumor immunotherapy response [23]. However, the immunological mechanisms by which inosine prevents colorectal cancer remain unknown. The purpose of this study was to look at the effects of inosine on macrophage polarization and its function in suppressing CRC progression in the tumor microenvironment.

## 2. Results

### 2.1. Inosine Promoted Macrophage M1 Phenotypic Polarization

**Figure 1:**
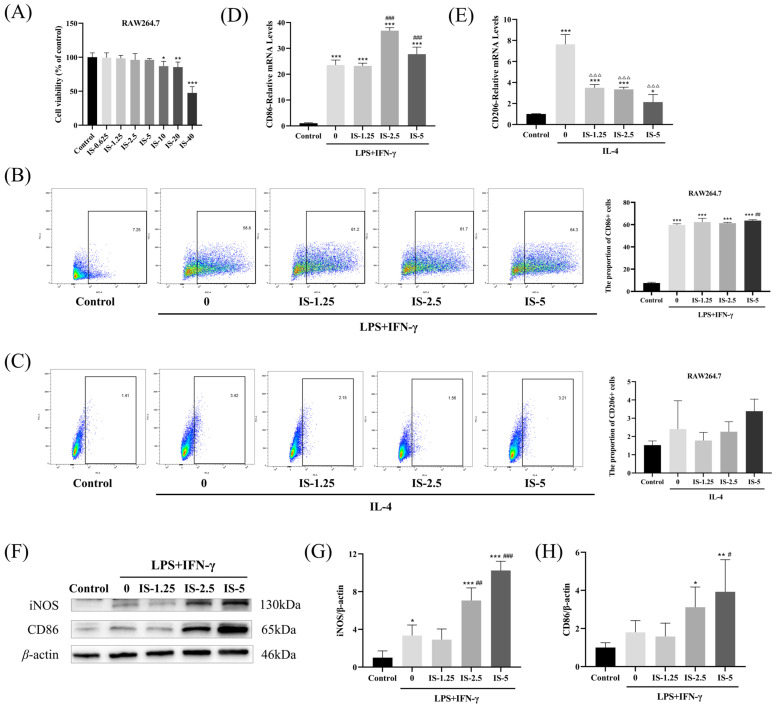
Inosine promoted macrophage polarization toward the M1 phenotype. (**A**) RAW264.7 cells were treated with inosine (0–40 mM) for 24 h; then, an MTT assay was performed to observe cell viability. RAW264.7 cells were administrated with inosine (1.25, 2.5, and 5 mM) in the absence or presence of LPS+IFN-γ or IL-4. (**B**,**C**) The proportions of CD86 and CD206-positive cells were determined by a flow cytometer. (**D**,**E**) The expression levels of CD86 and CD206 mRNA were detected by RT-qPCR. (**F**–**H**) Levels of CD86 and iNOS were measured by WB. Compared to the control group: * *p* < 0.05, ** *p* < 0.01, *** *p* < 0.001; Compared with the LPS+IFN-γ induced M1 group: ^#^ *p* < 0.05, ^##^ *p* < 0.01, ^###^ *p* < 0.001; Compared with the IL-4 induced M2 group: ^ΔΔΔ^ *p* < 0.001.

We explored the effect of inosine on macrophage polarization using RAW264.7 cells. Inosine had no inhibitory effect on the viability of the macrophage at concentrations below 10 mM (Figure 1A). Therefore, 1.25–5 mM inosine was selected for subsequent experiments.

RAW264.7 cells were stimulated with LPS+IFN-γ or IL-4 and varying inosine concentrations to observe changes in M1/M2 polarization. In the LPS+IFN-γ-induced M1 macrophage model, compared with the no-inosine group, there was a trend towards an increase in the proportion of CD86-positive cells in all experimental groups given inosine, with a significant increase observed at the highest inosine concentration (*p* < 0.01) (Figure 1B). However, there was less effect on IL-4-induced M2 macrophages, but it tended to increase in the high-dose group (Figure 1C). Meanwhile, as expected, inosine significantly increased mRNA and protein expression of M1 markers (*p* < 0.05) (Figure 1D,F–H). It had a significant inhibitory effect on the mRNA expression of M2 markers, but further validation is needed for their protein expression (Figure 1E). These results suggest that inosine promotes macrophage polarization toward the M1 phenotype.

### 2.2. Inosine Inhibited CT26 Cell Progression and Metastasis by Affecting Macrophage Polarization

**Figure 2:**
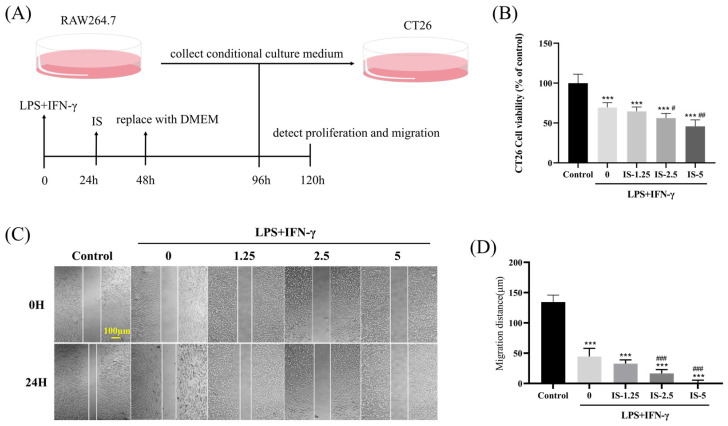
Inosine may exert an inhibitory effect on the progression and metastasis of CT26 cells by affecting macrophage polarization toward M1. (**A**) Schematic depicting the co-culture system and the experimental approach. (**B**) CT26 cells were treated for 24 h using a conditioned medium, and an MTT assay was used to measure the proliferation of CRC cells. (**C**) Wound healing was used to measure the metastasis of CRC cells (scale: 100 μm; 10×). (**D**) CT26 cells metastasis distance statistics. Compared to the control group: *** *p* < 0.001; Compared with the LPS+IFN-γ induced M1 group: ^#^ *p* < 0.05, ^##^ *p* < 0.01, ^###^ *p* < 0.001.

Further, based on the results of the previous experiments, we chose the promotional effect of inosine on M1 macrophages to explore its possible role in colorectal cancer cell proliferation and migration. CT26 cells are a colon cancer cell line derived from BALB/c mice and are primarily used as a model for tumor research. CT26 cells were co-cultured with a conditioned medium of M1 macrophages (with or without inosine) for 24 h, and changes in their proliferation and migration potential were assessed (Figure 2A). Due to the immune effect of macrophages, the co-culture could significantly inhibit the proliferation and migration of tumor cells (*p* < 0.05). Meanwhile, macrophages under the influence of inosine could enhance the inhibition of tumor cell proliferation (Figure 2B) and migration (Figure 2C,D) (*p* < 0.05). These findings suggested that inosine amplified the anti-tumor activity of M1 macrophages, further inhibiting the proliferation and migration of CRC cells.

### 2.3. Inosine Inhibited Solid Tumors of Colorectal Cancer

**Figure 3:**
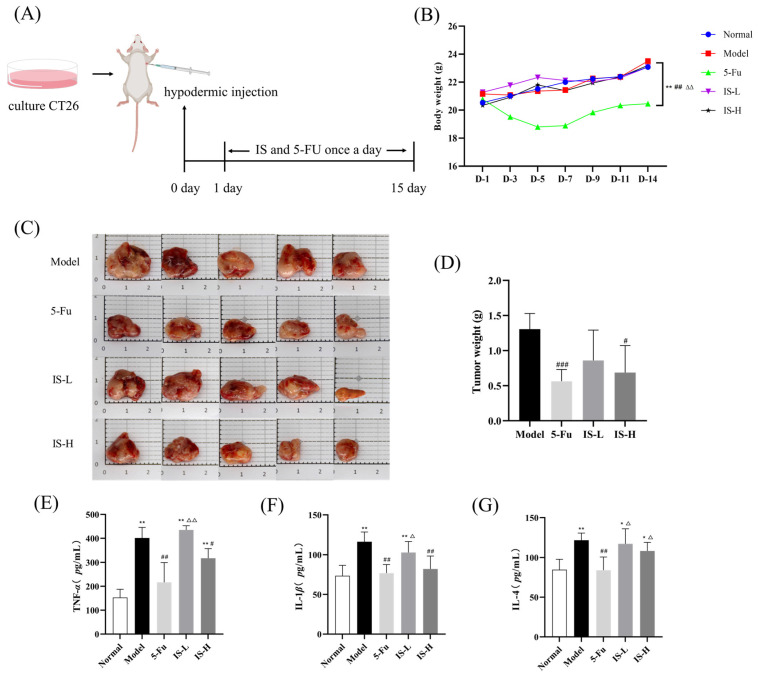
Anti-tumor efficacy of inosine in CT26 tumor-bearing mice. (**A**) Schematic diagram of mouse model establishment and administration. Mice were administered once daily for 14 days. (**B**) Mice were subjected to body weight measurements performed at regular intervals of 2 days. (**C**) Photographs of dissected mouse tumors at 15 days of modeling. (**D**) Evaluation of tumor weight. (**E**–**G**) Changes in serum levels of TNF-α, IL-1β, and IL-4 in mice of different groups. Note: 5-Fu are 5-Fu (12 mg/kg) groups. IS-L and IS-H are inosine low and high-dose (5 mg/kg and 50 mg/kg) groups, respectively. Compared to the normal group: * *p* < 0.05, ** *p* < 0.01; Compared with the model group: ^#^ *p* < 0.05, ^##^ *p* < 0.01, ^###^ *p* < 0.001; Compared with the 5-Fu group: ^△^ *p* < 0.05, ^△△^ *p* < 0.01.

Based on the results of the in vitro experiments, we further evaluated the effect of inosine on colorectal cancer solid tumors in vivo. 1 × 10^6^ CT26 cells were injected into the right axilla of BALB/c mice to establish a xenograft tumor model, and then the inosine and 5-Fluorouracil were administered by intraperitoneal injection (Figure 3A). 5-Fu is an essential chemotherapy drug widely used to treat colon cancer patients in the standard first-line treatment for several decades and has been employed as a positive control drug in preclinical animal studies [24]. During the experiment, mice in the normal control group had smooth and glossy fur, normal mobility, and dietary water intake. In contrast, the mice in the model group had dull hair color and showed slight hair-baring. After treatment, the hair color of the mice gradually recovered, with the 5-Fu group and the inosine high-dose group being the closest to the normal group. For the weight changes, except for the 5-Fu group with significant weight fluctuations, body weight changes remained stable across groups (Figure 3B). The results of the tumor inhibition rate showed that compared with the model group, the tumor weight of mice in the low and high-dose groups of inosine was reduced, of which the highest dose showed the most substantial reduction (*p* < 0.01), and the tumor inhibition rates were 34.09% and 47.39%. The overall tumor inhibition rate of inosine showed a dose-effect-dependent relationship, which intuitively showed the good tumor inhibitory activity of inosine (Figure 3C,D).

Changes in serum cytokine levels, including TNF-α, IL-1β, and IL-4, were determined by ELISA. The results indicated that the serum TNF-α, IL-1β, and IL-4 levels were significantly increased in the model group. The 5-Fu group suppressed this increase to a certain extent (*p* < 0.01). Inosine treatment also markedly decreased the upregulation in TNF-α and IL-1β levels in the serum of the model mouse (*p* < 0.05), and a similar trend was also observed in serum IL-4 levels, although there was no significant difference (Figure 3E–G).

### 2.4. Inosine Affected Ki-67 Expression in Colorectal Tumor Tissues

**Figure 4:**
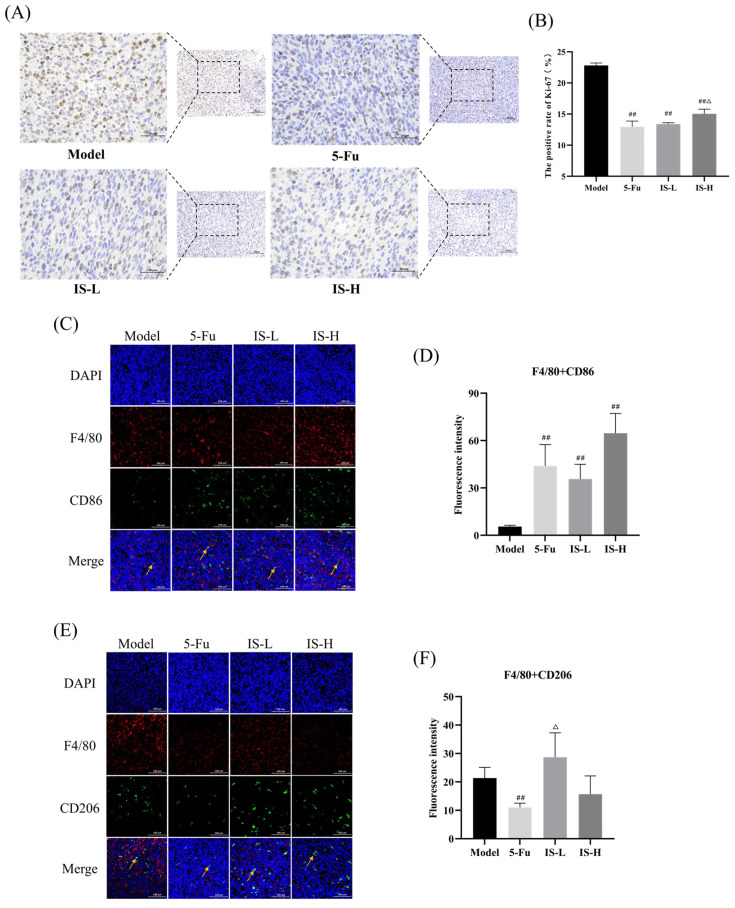
Effects of inosine on immune factors in the CT26 tumor microenvironment. (**A**) Effect of inosine on Ki-67 expression in tumor tissues (Scale: 100 μm; 400× and 200×). (**B**) Statistics of Ki-67 protein positive expression in tumor tissues (*n* = 3). (**C**) Fluorescence co-localization fluorogram of M1-type macrophage marker F4/80 + CD86 (scale: 100 μm; 200×). (**D**) F4/80 + CD86 expression statistics in tumor tissues. (**E**) M2 type macrophage marker F4/80 + CD206 fluorescence co-localization fluorogram (scale: 100 μm; 200×). (**F**) F4/80 + CD206 expression statistics in tumor tissues. Note: 5-Fu are 5-Fu (12 mg/kg) groups. IS-L and IS-H are inosine low and high-dose (5 mg/kg and 50 mg/kg) groups, respectively. Yellow arrows represent positive positions. Compared with the model group: ^##^ *p* < 0.01; Compared with the 5-Fu group: ^△^ *p* < 0.05.

Ki67, as a protein present in the nucleus, is associated with cell division and proliferative activity, and its elevated expression indicates enhanced cell division and proliferative activity. As shown in Figure 4A,B, both the positive drug and inosine administrated groups significantly reduced the expression of Ki-67 in CT26 colorectal tumor tissues compared to the model group (*p* < 0.01).

### 2.5. Inosine Promoted M1 Macrophage Anti-Tumor Effects in Colorectal Tumor Tissues

In the tumor microenvironment, macrophages mainly exhibit two activated morphologies, M1 and M2. F4/80 is a marker for macrophages, while CD86 and CD206 are the markers for M1 and M2 types of macrophages, respectively. Using immunofluorescence, F4/80 + CD86 (M1) and F4/80 + CD206 (M2) were double-stained and labeled to analyze the effect of inosine on M1 and M2-type macrophages in tumor tissues. The results were shown in Figure 4C–F, inosine significantly increased the expression of F4/80 + CD86 in a concentration-dependent manner compared to the model group (*p* < 0.01). The inosine high-dose group tended to decrease F4/80 + CD206 expression, while the inosine low-dose group tended to increase it. These results suggested that inosine promoted the polarization of macrophages toward the M1 phenotype in colorectal tumor tissues.

## 3. Discussion

Inosine, a purine nucleoside naturally present in the body, plays a key role in metabolism, energy production, and protein synthesis [23]. Clinically, it has been used to treat conditions like chronic hepatitis and Tourette’s syndrome in children [25]. Previous studies have shown that inosine enhanced effector T cell-mediated anti-tumor activity in animal models, contributing to tumor immunity [26]. Additionally, inosine could bind and inhibit the ubiquitin-activating enzyme UBA6, which enhanced the immunogenicity of tumors and improved responses to immunotherapy [23]. However, the current research on the anti-tumor aspects of inosine for immunomodulation is unclear in terms of whether it affects macrophage polarization.

In this study, we preliminarily evaluated the effect of inosine on the polarization of the M1/M2 phenotype of RAW264.7 macrophages in vitro while co-culturing them with colorectal cancer cells to affect colorectal cancer progression and metastasis. Further, the tumor inhibitory activity of inosine was explored using the CT26 colorectal tumor mouse model. Then, the expression of M1 and M2-type macrophages in tumor tissues was analyzed from the perspective of the tumor microenvironment.

The TME is the cellular environment in which tumor or cancer stem cells exist, including surrounding immune cells, blood vessels, extracellular matrix (ECM), fibroblasts, lymphocytes, bone marrow-derived inflammatory cells, and signaling molecules [27]. TAMs are the most prominent immune cell type in the TME and constitute the plastic and heterogeneous cell population of the TME, which can account for up to 50% of some solid tumors [28]. They promote the escape of tumor cells into the circulatory system and suppress anti-tumor immune mechanisms and responses [29]. In general, macrophages exhibit two distinct activation states: the pro-inflammatory and anti-tumorigenic classical activation phenotype (M1) and the anti-inflammatory and pro-tumorigenic activation phenotype (M2), which is involved in tissue repair and growth [30,31]. Typically, M1 macrophages are induced by LPS and IFN-γ, and they produce pro-inflammatory cytokines, including IL-6, TNF-α, and IL-1β, which possess potent anti-tumor effects [32,33]. In contrast, M2 macrophages are polarized by IL-4 and secrete anti-inflammatory cytokines, such as IL-10, promoting tumor progression [34].

We explored whether inosine regulated the polarization of macrophages by using RAW264.7 cells. Among the markers of M1-type macrophages, CD86 is a co-stimulatory molecule involved in T-cell activation and regulation of immune response. In addition to this, iNOS is a nitric oxide-producing enzyme that is important for antimicrobial and anti-tumor effects. They are classical biomarkers of macrophage M1 phenotype. Our data revealed that 5 mM inosine effectively increased the proportion of CD86+ cells and M1 macrophage markers, including the levels of CD86 mRNA and the expressions of CD86 and iNOS proteins. This suggested that inosine promoted macrophage polarization toward the M1 phenotype. CD206 is a classical M2 macrophage marker. Conversely, inosine inhibited the expression of the M2 macrophage marker CD206 at the mRNA level, although its impact on CD206-positive cell proportions was minimal. Thus, further research is needed to clarify inosine’s effect on M2 polarization of RAW264.7 macrophages.

Emerging evidence supports the notion that promoting M1 macrophage polarization can inhibit cancer progression and metastasis [35]. The role of inosine on CRC cell proliferation and metastasis is currently unknown. Therefore, we hypothesized that inosine may exert anti-tumor effects by modulating macrophage polarization in the TME. As expected, in our in vitro co-culture experiment of CT26 and RAW264.7 cells, inosine significantly inhibited CRC cell proliferation and metastasis, likely through the promotion of M1 macrophage polarization.

M1 polarized macrophages recruit new Th1 cells via chemokines CXCL9 and CXCL10 and produce pro-inflammatory cytokines, such as TNFα and IL-1β, IL6, IL12, and IL23 [36]. TNF-α is a functionally complex cytokine whose multiple roles depend on the local microenvironment. In malignant diseases, TNF-α may play a role in cancer therapy and contribute to the host response to tumors, but it may also be involved in cancer progression and spread. In several animal models, TNF-α contributes to malignant progression, and there is evidence that TNF-α may have an autocrine or paracrine role in human ovarian cancer [37]. IL-1β is considered to be the quintessential multifunctional cytokine, and its overexpression may contribute to tumorigenesis and promote tumor invasion. It has been shown that IL-1β expression is upregulated in many solid tumors, including melanoma, colon cancer, and lung cancer [38]. Inosine significantly reduced the expression of TNF-α and IL-1β compared to the model group. It was suggested that inosine might have inhibited tumor invasion and malignant progression by suppressing the overexpression of TNF-α and IL-1β secreted by M1 macrophages, which in turn inhibited tumor invasion and malignant progression.

This immunosuppressive state of M2 macrophages is initiated by Th2-derived cytokines such as IL4, IL10, IL13, transforming growth factor beta (TGFβ), prostaglandin E2 (PGE2), or colony-stimulating factor 1 (CSF1 or M-CSF). M2 macrophages lose their antigen-presenting capabilities and are involved in tissue remodeling, debris scavenging, and immune modulation [32]. Inosine significantly reduced IL-4 expression and thus decreased M2-induced polarization.

Ki-67, a nuclear protein associated with cell division and proliferation, is widely used as a marker to evaluate cell growth [39]. Our immunohistochemical analysis revealed that inosine dose-dependently reduced the expression of Ki-67 in CT26 colorectal tumor tissues, suggesting that inosine was able to inhibit the division and proliferation of colorectal tumor cells. Additionally, immunofluorescence results showed that inosine markedly increased the expression of F4/80+CD86, an M1 macrophage marker, in tumor tissues. Interestingly, the inosine group also tended to reduce the expression of F4/80+CD206, an M2 macrophage marker, suggesting that inosine may promote M1 polarization and enhance the anti-tumor effect of M1 macrophages.

## 4. Materials and Methods

### 4.1. Materials

Inosine (9HXZ-9DTA, 98%) was purchased from the China Academy of Food and Drug Administration. CD86/CD206-iF488-Tyramide (G1231), F4/80-iF647-Tyramide (G1232), RPMI-1640 medium (G4531) and DMEM high glucose medium (G4511) were purchased from Servicebio (Wuhan, China). Fetal Bovine Serum (FBS) was purchased from Cegrogen (German). Double antibiotic (penicillin and streptomycin, BL505A) was purchased from Biosharp (Beijing, China). APC Rat anti-Mouse CD86 (558703) was purchased from BD Pharmingen. PE Rat anti-Mouse CD206 (2696705) was purchased from Invitrogen (Carlsbad, CA, USA).

### 4.2. Cell-Based In Vitro Pharmacological Assays

#### 4.2.1. Cell Culture and Polarization Induction of Macrophages

Mouse colorectal cancer cell line CT26 and mouse monocyte macrophage leukemia cells RAW264.7 were purchased from the Typical Cultures Preservation Committee Cell Bank, Chinese Academy of Sciences. CT26 cells were cultured in RPMI-1640 medium containing 10% FBS and 1% double antibiotic in a 5% CO_2_ atmosphere at 37 °C. RAW264.7 cells were maintained in DMEM. For polarization induction, the macrophages were treated with 1 μg/mL LPS plus 20 ng/mL IFN-γ for 24 h to M1 polarization or 20 ng/mL IL-4 for 48 h to M2 polarization [40].

#### 4.2.2. Co-Culture Experiment

After RAW264.7 cells were polarized, they were treated with different concentrations of inosine for an additional 24 h. The conditioned medium was collected and subsequently used to co-culture with colorectal cancer cells, thereby establishing a macrophage-colorectal cancer co-culture system.

#### 4.2.3. Cell Viability

RAW264.7 cells were seeded into 96-well plates at 5000 cells/well. Following 24 h of incubation with various concentrations of inosine, cell proliferation was detected by adding 20 µL of MTT solution to each well. A microplate reader determined the OD value at 492 nm after four hours.

#### 4.2.4. Reverse Transcription Quantitative Polymerase Chain Reaction (RT-qPCR)

A SteadyPure quick RNA extraction kit (AG21023, Accurate Biology, Changsha, China) was applied to extract RNA from cells. The concentration of RNA was detected by a Full-wavelength Ultra-micro UV-Vis Spectrophotometer (Fc-3100, Life Real). The first complementary DNA (cDNA) strand was synthesized using the cqMAN Reverse Transcription System (AG11728, Accurate Biology, Changsha, China). Next, qPCR analyses (10 µL) were conducted according to the ratio of RNase-free water: 2x SYBR Green Pro Taq HS Premix (AG11701, Accurate Biology, Changsha, China): Forward primer: Reverse primer: cDNA = 3.6:5.0:0.2:0.2:1.0. The concentration of all primers was 100 μM. The relative RNA levels were estimated using a 2^−∆∆Ct^ method. Primer sequences that were used (Sangon Biotech, Shanghai, China) were listed in Table 1. GAPDH was used as an internal reference.

#### 4.2.5. Western Blot

Cells or tumor tissues were collected, and total proteins were extracted with RIPA buffer (Solarbio, Beijing, China). The Bicinchoninic Acid (BCA) method was applied to detect protein concentrations. Equivalent proteins were separated via 10% SDS-PAGE (PG212, Epizyme, Shanghai, China) and then transferred to PVDF membranes (IPVH00010, Millipore, MA, USA). Next, incubation with primary antibodies overnight was performed at 4 °C after the membranes were blocked with a 5% skim milk powder solution at room temperature for 1.5 h. Following rinsing in Tris Buffered Saline-Tween (TBST, G0004, Servicebio, Wuhan, China), the membranes were incubated for 2 h at room temperature with HRP-labeled secondary antibodies (1:5000). The developed membrane by chemiluminescence reagents (S6009M, UElandy, Suzhou, China) was subjected to band intensity quantification using ImageJ 1.8.0 software. Primary antibodies against CD86 (1:1000, 26903-1-AP, Proteintech, Wuhan, China), iNOS (1:300, 22226-1-AP, Proteintech, Wuhan, China), *β*-actin (1:2000, GB15003-100, Servicebio, Wuhan, China) and secondary antibody against HRP Goat Anti-Rabbit IgG (1:5000, AS014, ABclonal, Wuhan, China) were used, respectively.

#### 4.2.6. Macrophages Polarization Detection by Flow Cytometry

The treated cells were collected and prepared into single-cell suspension at least 1 × 10^6^/mL. After washing and blocking, cells were incubated with APC Rat anti-Mouse CD86 according to the manufacturer’s instructions. After binding with the fluorescent-labeled second antibody, fluorescence intensity was measured by flow cytometer (BD FACSCanto II).

#### 4.2.7. Wound Healing Assay

Approximately 1 × 10^6^ CT26 cells were plated into each well of the 6-well plate. When cell density reached about 90%, a 10 μL sterile pipette was applied to create scratches on the cell monolayer. Open wound areas were photographed using a microscope at 0 or 24 h. Finally, migration distance relative to the initial scratch was analyzed using ImageJ 1.8.0 software.

### 4.3. Animal

#### 4.3.1. Experimental Models and Drug Administration

All animal experiments in this study were conducted at Dali University, following the guidelines for the care and use of laboratory animals. The Animal Ethics Committee of Dali University evaluated and approved all experimental protocols. 25 Specific Pathogen Free(SPF)-grade male BALB/c mice (18–22 g, License No. SCXK (Beijing) 2019-0008) were acclimatized for 7 days with ad libitum access to food and water. The remaining 20 mice were anesthetized with isoflurane except for 5 from the normal group. Then, the mice were sterilized subcutaneously in the right axilla with alcohol. In order to create a subcutaneous CT26 colorectal tumor model in BALB/c mice, 100 µL of CT26 cells with a concentration of roughly 1 × 10^7^ cells/mL were extracted using a 1 mL syringe and subcutaneously injected uniformly for 1.5 to 2.0 cm in the direction of the parallel skin. After 24 h, the mice were randomly divided into five groups: normal, model, 5-Fu, and IS low- and high-dose groups. The normal and model groups received equal volumes of 0.9% NaCl, the 5-Fu group received 12 mg/kg 5-Fu, and the IS low- and high-dose groups were treated with 5 and 50 mg/kg inosine, respectively. Treatments were administered once daily for 14 days.

#### 4.3.2. Tumor Inhibition Rate and Tumor Histology in Mice

24 h after the final treatment, the mice were euthanized, and tumor tissues were excised, weighed, and photographed. Tumor inhibition rates were calculated as follows:

Tumor inhibition rate (%) = (1 − mean tumor weight of the treatment group/mean tumor weight of the model group) × 100.

Tumor tissues were fixed in 10% formalin, embedded, sectioned, and stained with hematoxylin and eosin (H&E) for histological analysis. And the pathological and histological changes in mice were finally observed under the electron microscope.

#### 4.3.3. ELISA Assay

At the end of the experiments, the mouse blood samples were then collected and placed at room temperature for 1 h to allow the blood samples to coagulate. Thereafter, the samples were centrifuged at 4 °C. Serum samples were aspirated and preserved at −80 °C. Changes in cytokine levels, including TNF-α, IL-1β, and IL-4, were detected by using relative ELISA kits according to the manufacturer’s instructions.

#### 4.3.4. Immunohistochemical Assay

Ki-67 expression in tumor tissues was detected using immunohistochemistry. After deparaffinization and antigen retrieval, sections were blocked, incubated with primary and secondary antibodies, and developed with DAB chromogenic solution. Hematoxylin was used for nuclear counterstaining, and sections were dehydrated and sealed for observation.

#### 4.3.5. Immunofluorescence Assay

Paraffin-embedded tumor sections were deparaffinized, subjected to antigen retrieval, and blocked with serum. Primary antibodies were applied overnight at 4 °C, followed by secondary antibody incubation at room temperature for 50 min. After Phosphate Buffered Saline (PBS) washes, DAPI staining was performed. Subsequently, an autofluorescence quencher was added for 5 min, and the sections were rinsed under running water for 10 min and blocked. Finally, sections were observed under a fluorescence microscope, and mean fluorescence intensity (MFI) was quantified using ImageJ 1.8.0 software.

### 4.4. Statistical Analysis

All data were analyzed using SPSS 23.0 software. Comparisons between two groups were made using a t-test, while one-way ANOVA was applied for comparisons among multiple groups. *p* < 0.05 indicated a statistically significant difference (* *p* < 0.05, ** *p* < 0.01, *** *p* < 0.001). Measurement data were presented as mean ± SEM.

## 5. Conclusions

Our findings suggested that inosine significantly increased M1 macrophage markers CD86 and iNOS in the tumor microenvironment and enhanced the anti-tumor activity of M1 macrophages, effectively inhibiting CRC progression and metastasis potential. In vivo, inosine had significant tumor inhibitory activity. It also significantly reduced the expression of Ki-67 and promoted the polarization of M1 macrophages.

## Figures and Tables

**Table 1:** The gene-specific primer sequences.

Primer Name	Forward Primer	Reverse Primer
CD86	AGCACTATTTGGGCACAGAGAAAC	GTGAAGTCGTAGAGTCCAGTTGTTC
CD206	CCTGAACAGCAACTTGACCAATAATG	GTTCTCCAGTAGCCATCAACATCC
GAPDH	GCAAATTCAACGGCACAGTCAAG	TCGCTCCTGGAAGATGGTGATG

## Data Availability

The original contributions presented in this study are included in the article. Further inquiries can be directed to the corresponding authors.
